# The *Candida* Genome Database: The new homology information page highlights protein similarity and phylogeny

**DOI:** 10.1093/nar/gkt1046

**Published:** 2013-10-31

**Authors:** Jonathan Binkley, Martha B. Arnaud, Diane O. Inglis, Marek S. Skrzypek, Prachi Shah, Farrell Wymore, Gail Binkley, Stuart R. Miyasato, Matt Simison, Gavin Sherlock

**Affiliations:** Department of Genetics, Stanford University Medical School, Stanford, CA 94305-5120, USA

## Abstract

The *Candida* Genome Database (CGD, http://www.candidagenome.org/) is a freely available online resource that provides gene, protein and sequence information for multiple *Candida* species, along with web-based tools for accessing, analyzing and exploring these data. The goal of CGD is to facilitate and accelerate research into *Candida* pathogenesis and biology. The CGD Web site is organized around Locus pages, which display information collected about individual genes. Locus pages have multiple tabs for accessing different types of information; the default Summary tab provides an overview of the gene name, aliases, phenotype and Gene Ontology curation, whereas other tabs display more in-depth information, including protein product details for coding genes, notes on changes to the sequence or structure of the gene and a comprehensive reference list. Here, in this update to previous NAR Database articles featuring CGD, we describe a new tab that we have added to the Locus page, entitled the Homology Information tab, which displays phylogeny and gene similarity information for each locus.

## INTRODUCTION

The *Candida* Genome Database (CGD, http://www.candidagenome.org/) is a freely available online resource, modeled after the *Saccharomyces* Genome Database [SGD, http://www.yeastgenome.org; (1)], which collects, organizes and distributes *Candida* gene, protein and sequence information to the fungal research community. CGD also provides web-based tools for data visualization and analysis.

Within the genus *Candida*, *Candida albicans* is the best-studied organism, as it is a common commensal within mammalian hosts as well as a pathogen that causes painful opportunistic mucosal infections in otherwise healthy individuals and causes severe and deadly bloodstream infections in the susceptible severely ill and/or immunocompromised patient population (2). This fungus exhibits a number of properties associated with the ability to invade host tissue, to resist the effects of antifungal therapeutic drugs and the human immune system and to alternately cause disease or coexist with the host as a commensal, including the ability to grow in multiple morphological forms and to switch between them, and the ability to grow as drug-resistant biofilms (3–7). The interplay between the fungus and the host immune system is complex; even the commensal state may not be as harmless as it has been assumed to be, as *Candida* interaction within the gut may set up a self-reinforcing inflammatory cycle (8,9). *C. albicans* is not the only disease-causing species in the genus; of serious concern is an emerging clinical prevalence of non-*albicans Candida* species (10–12). Among these, *Candida tropicalis* is common, virulent and increasingly resistant to antifungal therapy (13), *Candida parapsilosis* is observed to cause severe infections in neonates (14) and *Candida glabrata* exhibits a notable ability to evade the immune system and survive after cellular engulfment, along with resistance to antifungal treatment (15–17). Much remains to be understood before we can control and mitigate the pathology and morbidity associated with *Candida* infections (8).

### Multispecies information in CGD

In 2004, CGD began as a community resource containing curated information for a single species, *C. albicans* (18). Recognizing the research community’s need for a centralized repository for accurate and up-to-date research data about all of the medically important *Candida* species, we have significantly expanded the scope of CGD (19). We now perform manual curation of the scientific literature pertaining not only to *C. albicans*, but also to *C. glabrata*, *C. parapsilosis* and our most recently added species, *Candida dubliniensis*. For each of these species, we collect gene names and aliases, write descriptions to summarize the most important characteristics of each gene product, collect mutant phenotypes and assign relevant terms from the Gene Ontology, which is a structured vocabulary describing the precise function, cellular location and biological context in which each gene product acts (Table 1). We assemble comprehensive reference lists of all of the citations concerning each gene, and for those genes with sufficient literature, we also write free-text bullet-point summary notes.

**Table 1.** CGD multispecies curation statistics

Species	Verified genes	Uncharacterized genes	Manually curated GO	Orthology-based GO	Domain-based GO	Phenotypes
*Candida albicans* SC5314	1504	4558	8555	22 496	5041	15 205
*Candida dubliniensis* CD36	13	5849	33	27 765	5271	56
*Candida glabrata* CBS138	207	5006	669	27 150	4434	659
*Candida parapsilosis* CDC 317	25	5812	62	27 155	5351	35

We currently perform manual literature curation for four species; this set of reference genomes comprises *C. albicans* SC5314, *C. glabrata* CBS138, *C. dubliniensis* CD36 and *C. parapsilosis* CDC 317. We provide sequence files and protein domain files for an additional seven strains, covering 11 genomes and 10 species in total: *C. albicans* SC5314, *C. albicans* WO-1, *C. dubliniensis* CD36, *C. glabrata* CBS138, *C. guilliermondii* ATCC 6260, *C. lusitaniae* ATCC 42720, *C. orthopsilosis* Co 90-125, *C. parapsilosis* CDC317, *C. tropicalis* MYA-3404, *D. hansenii* CBS767 and *L. elongisporus* NRLL YB-4239. Within curated species, we define a gene to be ‘Verified’ if there is some experimental evidence for function (e.g. a mutant phenotype, or enzymatic activity); otherwise, we define the gene to be ‘Uncharacterized.’

For an even broader set of species and strains, including species that are not yet being actively curated, we generate and provide a suite of sequence files in consistent format. The standard sequence file set comprises FASTA files of chromosomes/contigs, coding and genomic sequence of annotated features with and without flanking regions, intergenic regions and protein sequences. We also perform InterProScan analysis (20) of each genome and make downloadable files available with predicted protein domains and motifs. We make sequence files and InterProScan analyses available for *C. albicans* SC5314, *C. albicans* WO-1, *C. dubliniensis* CD36, *C. glabrata* CBS138, *Candida guilliermondii* ATCC 6260, *Candida lusitaniae* ATCC 42720, *Candida orthopsilosis* Co 90-125, *C. parapsilosis* CDC317, *C. tropicalis* MYA-3404, *Debaryomyces hansenii* CBS767 and *Lodderomyces elongisporus* NRLL YB-4239.

The CGD web interface is organized around our gene-focused Locus pages, on which information collected about individual genes is displayed; Locus pages comprise a summary view along with several additional tabs that display more detailed information, including phenotype details, Gene Ontology term curation, protein product details for coding genes, notes on changes to the sequence or structure of the gene and a comprehensive reference list. Our newest addition to the Locus page is the Homology Information tab, a place where phylogeny- and similarity-related data may be examined and evaluated.

## THE NEW CGD HOMOLOGY INFORMATION TAB

The CGD Homology Information page allows users to explore relatedness among gene products across *Candida* species and between *Candida* and more distantly related organisms. The value of this is several-fold. Among species within the *Candida* genus, there are differences in pathogenicity and the underlying biology, which comparative biological approaches may help elucidate. Comparison with organisms further afield can shed light on possible functions of gene products that have not been directly characterized in *Candida*.

### Orthologs on the CGD homology information page

**Figure 1.**
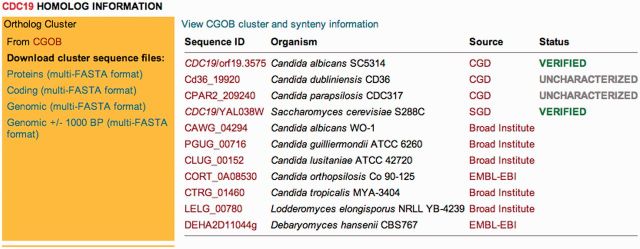
Ortholog cluster and Gene Links on the CGD Homology Information tab. The section entitled ‘Ortholog Cluster’ contains a link to the corresponding CGOB page for the ortholog group. Each of the clustered sequences is listed with links to its source database (e.g. the SGD, the Broad Institute, EMBL-EBI or CGD itself). The experimental status of each CGD and SGD gene is also given in this section, indicating whether there is published evidence for the existence of the gene as a functional entity. Links are also provided to download sequence files. In cases where a CGD-curated species is not included in the ortholog cluster but nevertheless has a high-scoring BLAST hit, that sequence is included in the next section of the page, entitled ‘Best hits in CGD species.’ Additional related proteins, from both more distantly related fungi and from non-fungal species, are listed along with links to gene information pages at their respective organism database sites.

In CGD, we use the ortholog groupings, or clusters, defined by Geraldine Butler’s group at the Conway Institute, University College Dublin, for their *Candida* Gene Order Browser tool (CGOB, http://cgob3.ucd.ie/) (21). Based on the framework developed for the Yeast Gene Order Browser (YGOB) (22), CGOB displays a graphical alignment of each ortholog cluster and its neighboring genes, allowing at-a-glance evaluation of the synteny across related species. At the top of each gene’s new Homology page in CGD, there is a section entitled ‘Ortholog Cluster’ with links to the corresponding CGOB page for that gene’s ortholog cluster. A list of all cluster sequences is also provided in this section, with links to an information page for each sequence from its source database (Figure 1). Genes from curated species in CGD are at the top of this list, with links to their respective Locus pages. If the cluster includes a sequence from *Saccharomyces cerevisiae*, that is listed next, with links to its Locus page at the SGD, followed by the remaining cluster sequences. The experimental status of each CGD and SGD gene is also given in this section, indicating whether there is evidence for its existence (‘Verified’ status) or not (‘Uncharacterized’ status), or are likely to be spurious [‘Dubious’ status, which has only been assigned to genes from *C. albicans,* see analysis published in (23)]. In the margin to the left of the ortholog list, we provide options for downloading sequence files in multiple-FASTA format: protein sequences, coding DNA sequences, genomic DNA sequences and genomic DNA sequences with the flanking 1000 bases upstream and downstream, for all of the members of the ortholog cluster. In cases where a CGD-curated species is not included in the ortholog cluster but nevertheless has a high-scoring BLAST hit, that sequence is included in the next section of the page, entitled ‘Best hits in CGD species’.

The sections of the CGD Homology page for orthologs and best hits in other species provide link-outs to information about related proteins in more distantly related species, including other curated model organism databases that provide gene-specific information. Orthologs from fungal organisms outside of the scope of CGOB are determined using the InParanoid program (http://inparanoid.sbc.su.se/). We link to *Aspergillus nidulans* genes at the *Aspergillus* Genome Database [AspGD; http://www.aspgd.org; (24)], *Schizosaccharomyces pombe* genes at PomBase [http://www.pombase.org; (25)] and *Neurospora crassa* genes at the Broad Institute (http://www.broadinstitute.org/annotation/genome/neurospora/). In cases where no ortholog is found in these species, top-scoring BLAST hits (if any) are listed. We also provide reciprocal best BLAST hits to genes from species outside of the fungi: *Dictyostelium discoideum* genes at dictyBase [dictybase.org; (26)], *Mus musculus* genes at Mouse Genome Database [MGD; http://www.informatics.jax.org; (27)] and *Rattus norvegicus* genes at Rat Genome Database [RGD; rgd.mcw.edu; (28)].

### Phylogenetic tree display

**Figure 2.**
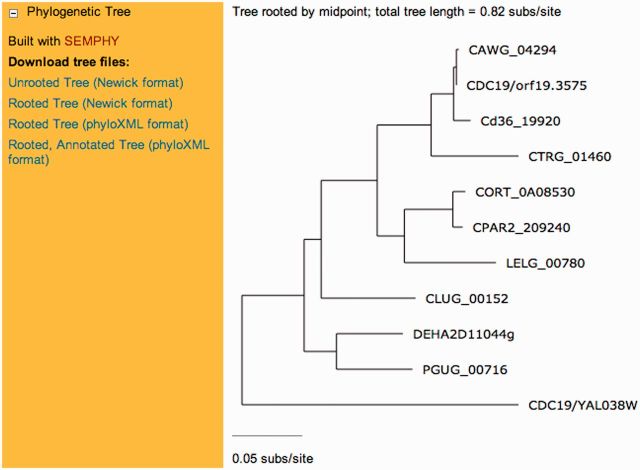
Phylogenetic Tree Display on the CGD Homology Information tab. The phylogenetic trees are computed from the protein multiple sequence alignment for each ortholog cluster, using the SEMPHY program (29). The species name is displayed in a hover box when the cursor is placed above the gene name, and the full species and gene names are also listed directly above the tree in the Ortholog Cluster section of the page. This section of the Homology Information page may be hidden or expanded using the small plus-or-minus glyph located to the left of the header in the gold-colored sidebar.

The Phylogenetic Tree display on the Homology Information tab provides a graphical illustration of the relatedness of the orthologs within the cluster (Figure 2). Trees are computed from the protein multiple sequence alignment (see later) for each cluster, using SEMPHY (29), and displayed using jsPhyloSVG (30). The length of the horizontal lines in the tree indicates the evolutionary distance (in substitutions per site) between sequences, which is proportional to the divergence time since the last common ancestor. The ‘total tree length’, or sum of all branch lengths in the tree, is given above the tree. This metric provides an estimate of the overall level of conservation within the ortholog cluster, with higher values indicating more variation (less conservation). Hovering the mouse cursor over the sequence IDs at the leaves of the tree reveals the host species. In addition to the graphical view, we provide tree data as downloadable files in Newick (see http://evolution.genetics.washington.edu/phylip/newicktree.html) and PhyloXML format (31). The Phylogenetic Tree section of the Homology Information tab may be hidden or expanded using the small glyph to the left of the header in the gold-colored sidebar.

### Alignments on the homology information page

**Figure 3.**
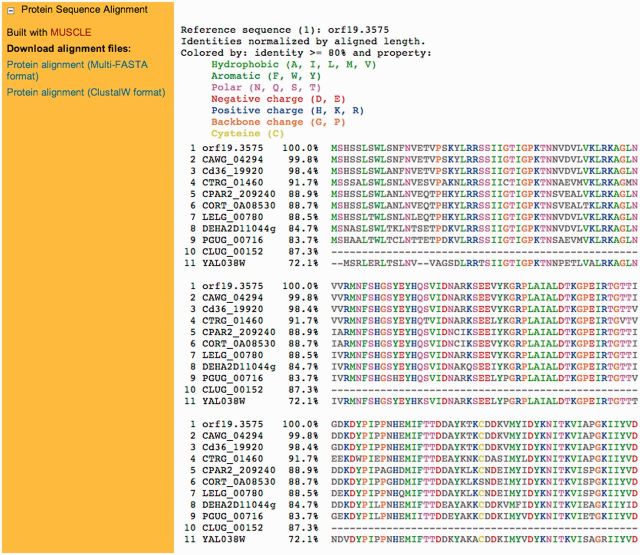
Protein Alignment Display on the CGD Homology Information tab. The Protein Sequence Alignment is a decorated multiple sequence alignment of the members of the ortholog cluster, generated using MUSCLE (32). The alignment display is generated with MView (33). The overall percentage identity to the reference sequence is displayed adjacent to the gene name. Alignment columns with <80% identity to the reference are displayed in black font. In columns with >80% consensus, the residues are color-coded by physicochemical properties as follows: hydrophobic residues (A, I, L, M, V) in light green, aromatic residues (F, W, Y) in dark green, polar residues (N, Q, S, T) in pink, residues with negative charge (D, E) in red, residues with positive charge (H, K, R) in blue, residues associated with backbone change (G, P) in red and cysteines (C) in yellow. A nucleotide alignment of the coding sequence is displayed below the protein alignment, with purine bases (A, G) color-coded in red and pyrimidines (C, T) displayed in blue. Like the Phylogenetic Tree, each sequence alignment may be hidden or expanded using the small plus-or-minus glyph located to the left of the header in the gold-colored sidebar.

The Protein Sequence Alignment section displays a decorated multiple sequence alignment of the peptide sequences (conceptual translation) of the genes within the ortholog cluster (Figure 3). Alignments are generated using the MUSCLE program (32), and the alignment display is generated by MView (33). The overall percentage identity, as compared with the reference sequence (protein sequence from the gene and species being viewed in CGD), is displayed next to the gene name. The alignment columns with <80% identity to the reference are displayed in black font. At positions with >80% identity, the residues are color-coded to indicate distinct physicochemical properties (e.g. hydrophobic residues are displayed in green font and negatively charged in red font). Coding sequence alignments are also displayed; these nucleotide alignments are generated directly from the protein sequence alignment, rather than by an independent alignment process; i.e. by substituting each amino acid from each protein sequence in the alignment with the corresponding triplet codon from the coding DNA sequence. Coding sequence alignments are also color-coded: alignment columns with ≥80% identity are colored red for purine bases or blue for pyrimidines. We provided these alignments for download in either multiple-FASTA or ClustalW format.

## CONCLUSIONS AND FUTURE DIRECTIONS

The CGD Homology Information tab provides a new resource for *Candida* homology and phylogeny data, with intuitive graphics and sequence retrieval options. In the future, we will provide quantification of conservation on a per-residue basis, and visualization tools to present these metrics for evaluation in the context of phylogeny, to provide an at-a-glance picture of evolutionary constraint, an indication of functional importance, at each position along the sequence. As more *Candida* genomes are sequenced, we will also provide additional analysis and graphical displays of polymorphism, including SNPs, indels, translocations and expansion of sequence repeats.

CGD is a freely available public community resource. Our ongoing mission is to serve the research needs of the scientific community studying *Candida* biology and pathogenesis, to thereby facilitate research progress and, ultimately, to have a positive impact on human health. CGD welcomes your feedback and suggestions; our curatorial staff can be reached by email at candida-curator@lists.stanford.edu.

## FUNDING

The National Institute of Dental and Craniofacial Research at the US National Institutes of Health [R01 DE015873]. Funding for open access charge: [R01 DE015873].

*Conflict of interest statement.* None declared.
